# Robust replication of associations across patient-mediated and provider-sourced EHR data in the *All of Us* research program

**DOI:** 10.1038/s44482-026-00032-8

**Published:** 2026-09-07

**Authors:** Chenjie Zeng, Bennett J. Waxse, Joshua C. Denny

**Affiliations:** 1 National Human Genome Research Institute, National Institutes of Health, Bethesda, MD USA; 2 National Institute of Allergy and Infectious Diseases, National Institutes of Health, Bethesda, MD USA; 3 All of Us Research Program, National Institutes of Health, Bethesda, MD USA

**Keywords:** Computational biology and bioinformatics, Diseases, Genetics, Health care, Medical research

## Abstract

The *All of Us* Research Program is assembling a nationwide cohort with electronic health record (EHR) resources through two complementary pathways: healthcare provider organization (HPO)-sourced EHRs and patient-mediated EHR (PME) contributed through patient portal linkages. The comparative research utility of these two data sources has not been systematically evaluated. Here, we compared PME and HPO EHRs with respect to disease prevalence, phenotype–phenotype associations, and replication of established genotype-phenotype associations using data from 19,703 PME and 373,887 HPO participants. We benchmarked disease prevalence against national estimates, conducted phenome-wide association studies for 10 commonly studied diseases, and tested replication of more than 5000 established genotype-phenotype associations across multiple ancestral groups. Disease prevalence was consistently lower in PME than in HPO, although prevalence of most diseases in both cohorts exceeded national estimates. Both data sources reproduced known phenotype–phenotype associations and showed moderate-to-strong concordance in effect sizes across the phenome. The overall genotype-phenotype replication rate was 49.1% (5399/10,999) in HPO and 5.9% (381/6482) in PME across ancestral groups, with effect sizes strongly correlated among well-powered associations (*R* = 0.84, *P* < 0.001). To disentangle the impact of sample size from data quality, we performed 1:1 propensity score matching. After matching, the replication gap in genotype-phenotype associations narrowed from 8.3-fold to 1.3-fold, with equivalent replication rates among adequately powered associations and strongly concordant effect sizes; comorbidity patterns were also consistent across all 10 diseases tested. These findings demonstrate that both data sources are valuable for clinical and genomic research and can inform other cohorts integrating provider-derived and patient-mediated EHRs.

## Introduction

The widespread adoption of electronic health records (EHR) has enabled large-scale investigations of disease patterns, treatment outcomes, and population health^1^. The 21st Century Cures Act established patients’ right to access the entirety of their EHRs through application programming interfaces^2^. This mandate helped catalyze the adoption of Health Level Seven’s Fast Healthcare Interoperability Resources (FHIR)^3^, which has since become the dominant standard for enabling secure, app-based exchange of health data across EHR systems^4^. Longitudinal cohort studies leveraging EHRs have dramatically advanced health by identifying intervenable risk factors and uncovering novel insights into disease etiology. The *All of Us* Research Program (*All of Us*) represents an important effort in this domain, aiming to enroll at least one million participants who share multiple sources of data, including longitudinal EHRs^5^.

A primary challenge in EHR-based research in the US lies in data fragmentation created by the patchwork of different healthcare systems from which a patient may receive care over their lifetime^6^. To address this, *All of Us* employs multiple approaches to EHR collection. Up to the most recent data release, EHR data *in All of Us* has been sourced directly from healthcare provider organizations (HPO), which convert their EHR data into the Observational Medical Outcomes Partnership common data model. Given that HPOs can change EHR vendor systems over time, HPO-uploaded EHR data can include data from multiple different EHRs systems. The list of HPO sites and descriptions can be found at https://www.joinallofus.org/health-care-provider-organizations. With the most recent release of data from *All of Us* (CDR v8), another source of EHR data has been added: patient-mediated EHRs (PME), where individuals contribute their health information from multiple patient portals^5^. The PME approach, typically represented via FHIR but can also be shared in other formats such as Consolidated Clinical Document Architecture^7^, represents a patient-centric model that can facilitate comprehensive longitudinal records. Thus, participants can share data from multiple providers that may be parts of different health care systems, including across different locations. This dual collection strategy offers unique opportunities to assess the comparability and research utility of these different data sources.

Previous efforts have demonstrated the capacity of HPO- based EHRs to support discovery across different domains, from genetic associations to population health^8–12^. Nearly all EHR-based studies to date have utilized data sourced through mechanisms similar to HPOs. Little is known about the validity of PME data for research purposes, and few resources beyond *All of Us* provide the opportunity to directly compare the utility of PME and HPO EHRs. Moreover, prior analyses have suggested that *All of Us* participants are, on average, sicker than the general population^13,14^, raising the possibility that PME participants may differ in important ways that could affect research findings.

Understanding the phenomic profiles of participants and the quality of data from each source is essential for conducting rigorous research within *All of Us* and for researchers using PME data. This study aims to systematically evaluate and compare the research utility of PME-FHIR data with HPO-sourced EHR data within *All of Us*. We assessed these two sources of EHR data across three domains: (1) comparing disease prevalence estimates between data sources and benchmarking against national statistics^15^, (2) conducting phenome-wide association studies (PheWAS)^10,16^ for common and rare diseases to evaluate the consistency of phenotypic associations, and (3) testing the replication rates for established genotype-phenotype associations^17^. To assess the impact of sample size and baseline characteristics, we performed 1:1 propensity score matching on key demographic, socioeconomic and EHR documentation characteristics, selecting an HPO subset (*n* = 19,703) comparable to the full PME cohort. Through multimodal analyses, we aim to understand the research utility and limitations of each data source, providing guidance for researchers utilizing *All of Us* and advancing understanding of how different approaches to EHR data collection influence research validity and inform future studies using PME-type data.

## Results

### Participant characteristics

**Fig. 1:**
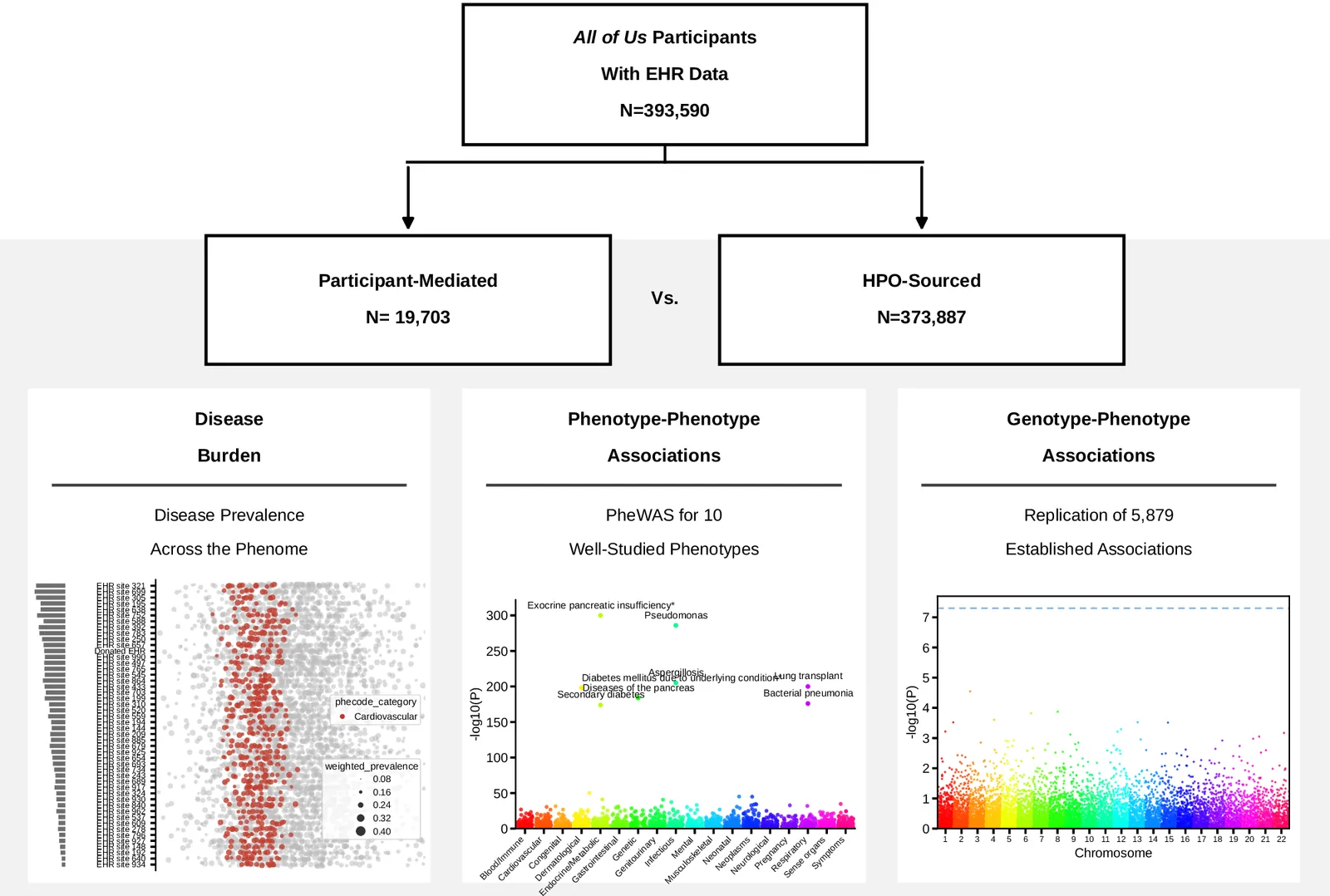
Study design for comparing patient-mediated and provider-sourced electronic health record data in the *All of Us* Research Program. Among 393,590 *All of Us* participants with electronic health record (EHR) data, records were classified by provenance into a patient-mediated group (N = 19,703) and an HPO-sourced group (N = 373,887). The two groups were compared across three analyses, illustrated schematically in the lower panels: disease burden, measured as disease prevalence across the phenome (left); phenotype–phenotype associations, evaluated by phenome-wide association study (PheWAS) for 10 well-studied phenotypes (middle); and genotype–phenotype associations, assessed by replication of more than 5000 established associations (right).

A total of 393,590 participants with EHRs were included (Fig. 1). Table 1 presents the baseline characteristics of participants in the PME and HPO cohorts. Compared to the HPO cohort, participants in the PME cohort were more likely to be White (75.8% vs 52.2%, *P* < 0.001), female (69.2% vs 60.4%, *P* < 0.001), and have health insurance (97.2% vs 92.6%, *P* < 0.001), while having a younger starting age in EHRs (38.5 vs 43.3 years, *P* < 0.001) and longer average EHR follow-up time (15.3 vs 12.3 years, *P* < 0.001). Additionally, the PME cohort participants were less likely to have whole-genome sequencing data (35.2% vs 83.2%, *P* < 0.001) in the current study.

**Table 1:** Baseline characteristics of participants by sources of EHRs in *All of Us*

Characteristics	HPO	PME
	373,887	19,703
Age at enrollment, in years (mean (SD))	51.79 (17.04)	52.10 (15.81)
Sex (%)		
Female	225,917 (60.4)	13,642 (69.2)
Male	143,874 (38.5)	5992 (30.4)
Self-reported race and ethnicity (%)		
White	195,343 (52.2)	14,930 (75.8)
Hispanic	70,243 (18.8)	1551 (7.9)
Black	66,315 (17.7)	881 (4.5)
Asian	10,770 (2.9)	569 (2.9)
AIAN	4409 (1.2)	84 (0.4)
MENA	2042 (0.5)	50 (0.3)
First age in EHRs, in years (mean (SD))	43.32 (17.17)	38.45 (18.40)
Length of EHR records, in years (median (IQR))	9.79 (6.20–16.68)	12.79 (7.06–20.99)
Had whole genome sequencing data	311,022 (83.2)	6939 (35.2)
Highest education attained (%)		
College degree and above	160,966 (43.1)	11,599 (58.9)
Less than College degree	203,889 (54.5)	8052 (40.9)
Annual household income (%)		
<25K	91,440 (24.5)	3167 (16.1)
25K–50K	55,525 (14.9)	3755 (19.1)
50k–75k	39,223 (10.5)	3167 (16.1)
75K–150K	68,054 (18.2)	5732 (29.1)
150K+	42,051 (11.2)	3130 (15.9)
Health insurance (%)		
No	22,456 (6.0)	442 (2.2)
Yes	346,246 (92.6)	19,144 (97.2)
BMI at enrollment, (mean (SD))	29.97 (7.65)	29.83 (7.87)
Smoking status (%)		
Current	61,491 (18.3)	2470 (14.2)
Former	60,013 (17.8)	4309 (24.8)
Never	215,065 (63.9)	10,577 (60.9)

*AIAN* American Indian or Alaska Native, *BMI* body mass index, *MENA* Middle Eastern or North African, *SD* standard deviation, *IQR* interquartile range.

### Disease prevalence comparison

**Fig. 2:**
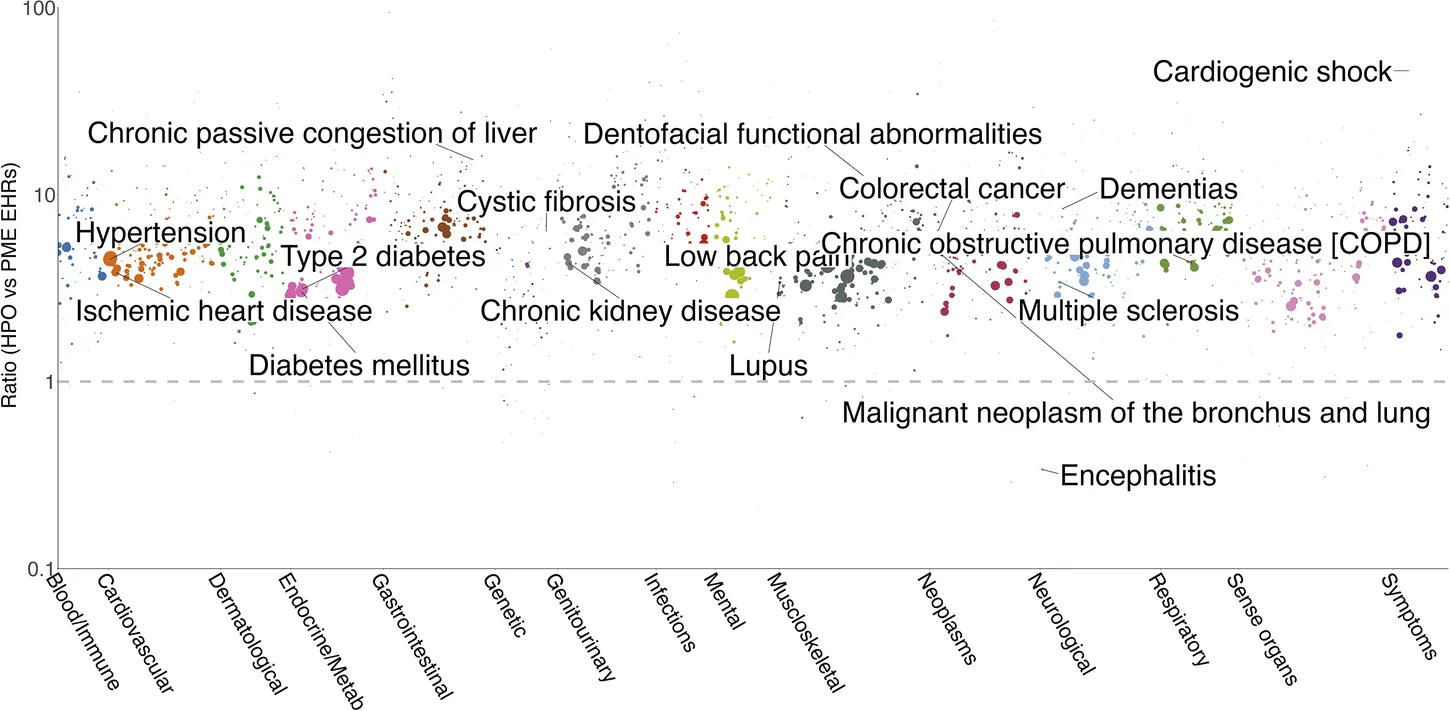
Prevalence ratios of conditions in HPO-sourced versus patient-mediated EHRs across disease categories. Each dot represents a phecode, colored by disease category and sized proportionally to the prevalence in the HPO cohort. The *y*-axis shows the prevalence ratio (HPO/PME) on a log10 scale; the gray horizontal line indicates a ratio of 1 (equal prevalence).

Figure 2 shows the comparison of disease prevalence by sources of EHRs and by sites. Disease prevalence was consistently lower in the PME cohort compared to the HPO cohort across nearly all phecode categories. Among 927 phecodes with at least 20 cases in both cohorts, the median prevalence ratio (HPO/PME, PR) was 5.12 (interquartile range: 3.90–6.99); (97.0%) had statistically significantly higher prevalence in the HPO cohort (PR > 1.1, *P* < 1.8 × 10^−5^), while only 2 (0.2%) conditions had a statistically significantly higher prevalence in the PME cohort (PR < 0.9, *P* < 1.8 × 10^−5^), mainly driven by encephalitis (PR = 0.34, *P* = 7.3 × 10^−12^). The remaining 26 (2.8%) showed no statistically significant difference between cohorts. The largest prevalence differences were observed for conditions including fracture of femur (PR = 34.5.), conditions in mothers complicating pregnancy (PR = 23.1), irregular menstrual cycle (PR = 23.0), and leiomyoma of uterus (PR = 19.6, Supplementary Table S1).

Compared to national estimates from the GBD study, both cohorts showed higher disease prevalence across most conditions, but the magnitude of this difference was greater in the HPO cohort than in the PME cohort (median PR vs GBD: 5.67 and 1.84 for HPO and PME, respectively, Supplementary Table S2, and Figs. S1 and 2).

### Phenotype-phenotype associations

**Fig. 3:**
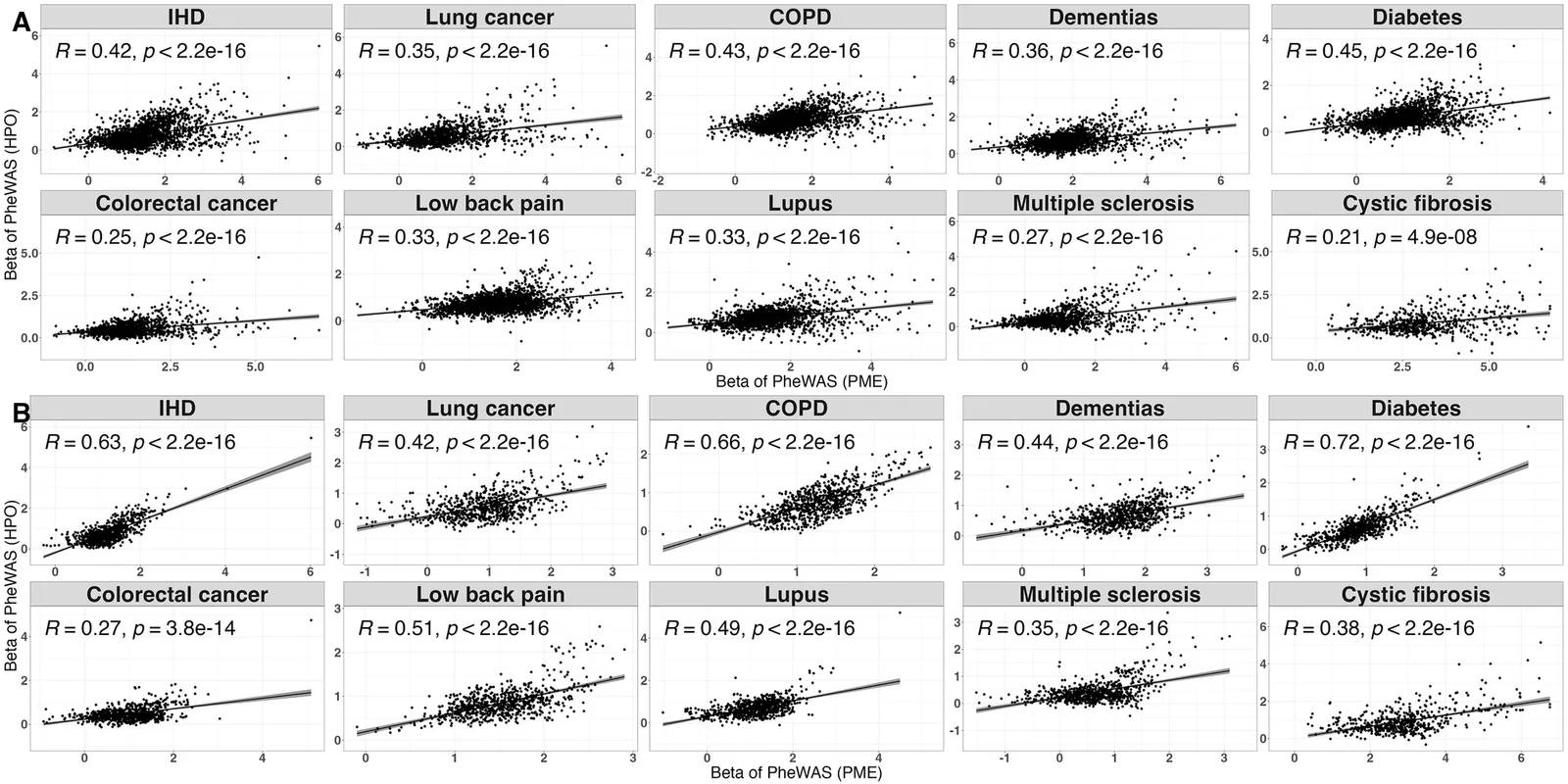
Correlation of phenotype-phenotype associations between PME and HPO. **A** Scatterplots showing the relationship between phecode effect-size estimates (β coefficients, i.e., log odds ratios) obtained from phenotype-anchored phenome-wide association studies (PheWAS) in the patient-mediated EHR cohort (PME; *x*-axis) and the healthcare provider organization-provided EHR cohort (HPO; *y*-axis). Each dot represents the β coefficient for one phecode tested for its association with the index disease shown in the panel title. For ischemic heart disease, lung cancer, COPD, dementias, diabetes, colorectal cancer, and low back pain, β coefficients were estimated by logistic regression; for lupus, multiple sclerosis, and cystic fibrosis, β coefficients were estimated by Firth logistic regression. All models were adjusted for age, sex at birth, self-reported race or ethnicity, length of EHR records, income, and education. Solid lines indicate the linear regression fit, with 95% confidence bands shaded in gray. Spearman rank correlation coefficients (*R*) and corresponding *P* values are reported in each panel. **B** Equivalent scatterplots restricted to phecode-index disease associations with at least 100 cases in both the PME and HPO cohorts. Each dot represents the β coefficient for one such association. All regression models, plotting conventions, and statistical reporting are identical to those in **A**.

Figure 3 shows the correlation of effect sizes from PheWAS for 10 selected diseases between the PME and HPO cohorts. Despite substantial differences in disease prevalence, we observed moderate concordance in disease-disease associations between the two cohorts. Correlation coefficients of effect sizes across the phenome ranged from 0.21 for cystic fibrosis to 0.45 for diabetes (all *P* < 0.001, Fig. 3A). When analyses were restricted to phenotypes with at least 100 cases in either cohort, correlations were consistently higher, ranging from 0.27 to 0.72, with the largest increases observed for common diseases, such as diabetes, ischemic heart disease (IHD), and chronic obstructive pulmonary disease (COPD) (*R* > 0.6, *P* < 0.001, Fig. 3B). Additionally, both cohorts replicated associations of well-known risk factors and comorbidities for each disease (Table S3). Table 2 presents selected known associations and their effect sizes in both cohorts. For common diseases like IHD and diabetes, established associations with conditions such as hypertension and obesity showed the same direction and similar magnitude in effect sizes in both cohorts. For rare diseases, known associations were detectable in both cohorts, but effect estimates were more variable in the PME cohort. Associations in both cohorts are presented in Table S3.

**Table 2:** Associations of selected known clinical correlates with diseases in participants stratified by sources of EHRs

Diseases	Known correlated clinical phenotypes	PME	HPO
		OR (95% CI)	OR (95% CI)
Colorectal cancer			
	Colorectal polyps	3.09 (1.88–5.07)	5.4 (5.08–5.75)
	Nicotine dependence	2.74 (1.62–4.62)	2.03 (1.91–2.15)
	Alcohol abuse and dependence	1.89 (0.59–6.06)	1.50 (1.36–1.64)
	Gastrointestinal inflammation	5.83 (3.50–9.70)	2.85 (2.69–3.02)
	Malnutrition and underweight	5.55 (2.95–10.44)	3.19 (3.00–3.39)
	Secondary malignant neoplasm	14.86 (6.45–34.22)	9.12 (8.52–9.77)
	Gastrointestinal obstruction	7.23 (3.74–13.97)	4.29 (4.01–4.58)
COPD			
	Nicotine dependence	8.86 (7.51–10.45)	7.27 (7.08–7.47)
	Bronchitis	14.6 (11.89–17.94)	5.61 (5.46–5.77)
	Respiratory infection	8.95 (7.47–10.71)	4.01 (3.91–4.12)
	Dyspnea	8.59 (7.18–10.27)	7.04 (6.85–7.22)
	Acute lower respiratory infection	10.66 (8.38–13.55)	5.68 (5.52–5.84)
	Atherosclerosis	5.82 (4.84–7.00)	4.02 (3.91–4.13)
	Ischemic heart disease	6.00 (4.96–7.26)	4.07 (3.96–4.18)
	Pneumonia	9.65 (7.53–12.36)	5.87 (5.71–6.03)
	Acute bronchitis	9.62 (7.48–12.38)	3.87 (3.75–3.99)
	Back pain	4.07 (3.48–4.76)	3.15 (3.06–3.24)
	Asthma	4.07 (3.42–4.85)	5.89 (5.74–6.05)
	Alcohol abuse and dependence	4.35 (3.21–5.88)	3.35 (3.24–3.47)
Cystic fibrosis			
	Ileus	673.78 (71.94–6310.81)	172.91 (141.86–210.76)
	Bronchiectasis	478.67 (45.92–4989.22)	66.55 (54.11–81.84)
	Gastrointestinal obstruction	77.85 (12.12–499.95)	53.54 (43.68–65.62)
	Pneumonia due to Pseudomonas	1778.78 (68.65–46,091.61)	181.59 (138.04–238.87)
	Pseudomonas	178.36 (15.02–2118.37)	55.1 (44.42–68.34)
	Aspergillosis	7505.97 (328.91–171,294.44)	89.8 (67.73–119.06)
	Lung transplant	1598.21 (72.91–35,033.14)	96.44 (71.97–129.23)
	Diseases of the pancreas	36.28 (5.52–238.55)	16.55 (13.72–19.95)
	Secondary diabetes	228.52 (21.38–2442.43)	25.70 (20.65–31.98)
	Bacterial pneumonia	156.96 (22.35–1102.5)	19.83 (16.17–24.31)
	Staphylococcus aureus	46.55 (4.37–496.13)	11.15 (8.95–13.88)
	Chronic sinusitis	37.17 (6.02–229.50)	6.33 (5.25–7.64)
	Asthma	9.50 (1.53–59.05)	4.40 (3.67–5.26)
	Chronic pancreatitis	98.69 (9.82–992.04)	10.2 (7.33–14.19)
	Acute pancreatitis	34.63 (3.56–337.16)	3.50 (2.48–4.94)
Dementia			
	Cerebrovascular insufficiency	27.94 (15.16–51.50)	5.99 (5.52–6.50)
	Stroke	14.61 (8.92–23.91)	4.63 (4.37–4.90)
	White matter disease	48.77 (23.04–103.25)	10.70 (9.6–11.91)
	Malnutrition and underweight	8.32 (5.18–13.38)	3.37 (3.20–3.55)
	Atherosclerosis	6.21 (4.11–9.40)	2.75 (2.62–2.90)
	Anxiety and anxiety disorders	5.35 (3.61–7.91)	3.91 (3.71–4.11)
	Sleep disorders	5.06 (3.38–7.57)	2.90 (2.76–3.05)
	Pneumonia	5.88 (3.45–10.01)	2.80 (2.65–2.94)
	Hypertension	5.20 (3.31–8.17)	3.31 (3.10–3.54)
	Diabetes mellitus	3.36 (2.27–4.99)	2.18 (2.07–2.29)
	Nicotine dependence	5.47 (3.69–8.10)	2.12 (2.02–2.23)
	Alcohol abuse and dependence	5.05 (2.64–9.64)	2.72 (2.54–2.91)
	Hearing impairment	4.82 (3.15–7.39)	1.85 (1.76–1.96)
	Hypercholesterolemia	5.06 (3.42–7.50)	1.71 (1.63–1.80)
Diabetes			
	Obesity	5.41 (4.93–5.94)	5.22 (5.13–5.32)
	Hypertension	5.62 (5.10–6.19)	8.41 (8.23–8.60)
	Hyperlipidemia	4.61 (4.18–5.09)	6.82 (6.67–6.96)
	Sleep apnea	4.15 (3.76–4.58)	3.59 (3.52–3.67)
	Obstructive sleep apnea	3.95 (3.56–4.37)	3.62 (3.54–3.69)
	Chronic liver disease	5.30 (4.56–6.15)	3.81 (3.72–3.90)
	Atherosclerosis	3.85 (3.38–4.39)	3.55 (3.47–3.62)
	Anemia	3.1 (2.78–3.46)	2.81 (2.76–2.86)
	Nicotine dependence	2.02 (1.79–2.27)	1.99 (1.95–2.03)
	Alcohol abuse and dependence	1.42 (1.10–1.84)	1.37 (1.33–1.42)
IHD			
	Chest pain	7.39 (6.36–8.59)	5.60 (5.48–5.73)
	Abnormalities of breathing	6.25 (5.41–7.23)	5.12 (5.01–5.24)
	Heart failure	14.13 (11.39–17.54)	12.86 (12.49–13.24)
	Hypertension	5.64 (4.87–6.53)	8.44 (8.20–8.69)
	Hyperlipidemia	6.16 (5.26–7.22)	6.97 (6.78–7.16)
	Hypercholesterolemia	4.11 (3.60–4.70)	3.22 (3.15–3.29)
	Diabetes mellitus	4.21 (3.66–4.83)	3.80 (3.72–3.88)
	Malaise and fatigue	4.42 (3.83–5.11)	2.98 (2.92–3.05)
	Chronic obstructive pulmonary disease	6.22 (5.14–7.51)	4.17 (4.07–4.29)
	Nicotine dependence	4.24 (3.64–4.94)	2.96 (2.9–3.03)
	Obesity	3.14 (2.74–3.59)	2.8 (2.74–2.86)
	Chronic kidney disease	3.74 (3.14–4.47)	4.61 (4.49–4.72)
	Ventricular fibrillation	26.46 (4.33–161.77)	30.79 (25.72–36.85)
	Alcohol abuse and dependence	2.29 (1.66–3.17)	1.83 (1.77–1.89)
Low back pain			
	Pain in joint	7.42 (6.71–8.20)	5.43 (5.33–5.53)
	Spondylosis	12.02 (10.62–13.61)	7.86 (7.69–8.03)
	Malaise and fatigue	6.77 (6.08–7.54)	3.24 (3.19–3.30)
	Mood disorders	3.62 (3.28–3.99)	3.20 (3.14–3.25)
	Nicotine dependence	3.63 (3.23–4.07)	2.08 (2.04–2.12)
	Alcohol abuse and dependence	3.05 (2.43–3.82)	2.02 (1.96–2.07)
Lung cancer			
	Chronic obstructive pulmonary disease	17.16 (9.97–29.54)	7.77 (7.26–8.32)
	Secondary malignant neoplasm	33.43 (15.97–70.02)	26.23 (24.49–28.10)
	Nicotine dependence	8.29 (4.96–13.84)	4.75 (4.44–5.09)
	Acute lower respiratory infection	3.49 (1.56–7.82)	2.38 (2.21–2.56)
	Underweight	6.22 (1.39–27.80)	3.96 (3.44–4.56)
	Alcohol abuse and dependence	3.62 (1.28–10.22)	2.07 (1.87–2.28)
Lupus			
	Rheumatoid arthritis and other inflammatory polyarthritis	12.00 (8.27–17.41)	10.35 (9.70–11.04)
	Sicca syndrome	18.88 (12.06–29.56)	13.21 (12.17–14.33)
	Hypopyon	18.04 (11.09–29.36)	10.68 (9.68–11.78)
	Rheumatoid arthritis	10.94 (7.24–16.55)	10.65 (9.94–11.40)
	Raynaud’s syndrome	14.67 (9.1–23.63)	13.08 (12.02–14.24)
	Myalgia	6.28 (4.52–8.74)	4.08 (3.84–4.33)
	Glomerular diseases	56.84 (26.82–120.48)	14.51 (13.37–15.75)
	Peripheral vascular disease	8.96 (5.89–13.61)	5.57 (5.19–5.97)
	Hyper–coagulability	10.89 (6.14–19.3)	11.67 (10.76–12.67)
	Rash and other nonspecific skin eruption	5.92 (3.78–9.29)	3.19 (3.00–3.39)
	Renal failure	4.39 (2.88–6.68)	3.71 (3.48–3.96)
	Nicotine dependence	1.98 (1.34–2.93)	1.75 (1.65–1.86)
Multiple sclerosis			
	Optic neuritis	104.51 (40.49–269.74)	61.69 (55.13–69.02)
	Neuromuscular dysfunction of bladder	27.46 (13.85–54.45)	28.12 (25.76–30.68)
	Abnormality of gait and mobility	7.54 (4.85–11.74)	6.03 (5.59–6.50)
	White matter disease	33.51 (14.75–76.10)	17.7 (15.65–20.03)
	Optic neuropathy	23.98 (11.02–52.21)	19.01 (17.32–20.87)
	Plegia and unspecified paralysis	29.95 (12.73–70.45)	14.81 (12.98–16.89)
	Disturbances of skin sensation	5.06 (3.32–7.69)	4.82 (4.49–5.17)
	Lack of coordination	11.86 (5.78–24.32)	8.13 (7.34–9.00)
	Paraplegia/Diplegia	41.04 (10.92–154.24)	24.37 (20.84–28.49)
	Myelitis	404.69 (40.30–4063.53)	73.99 (61.05–89.66)
	Encephalitis, myelitis and encephalomyelitis	7.15 (2.81–18.18)	28.74 (25.00–33.04)
	Cerebral palsy and other paralytic syndromes	8.97 (3.53–22.77)	6.87 (6.16–7.67)
	Optic atrophy	18.02 (6.92–46.88)	8.52 (7.52–9.66)
	Nicotine dependence	2.27 (1.48–3.49)	1.47 (1.36–1.58)

*OR* odds ratio, *CI* confidence interval, *COPD* chronic obstructive pulmonary disease, *IHD* ischemic heart disease, *PME* patient-mediated EHRs, *HPO* healthcare provider organization-sourced EHRs.

### Genotype-phenotype associations

**Fig. 4:**
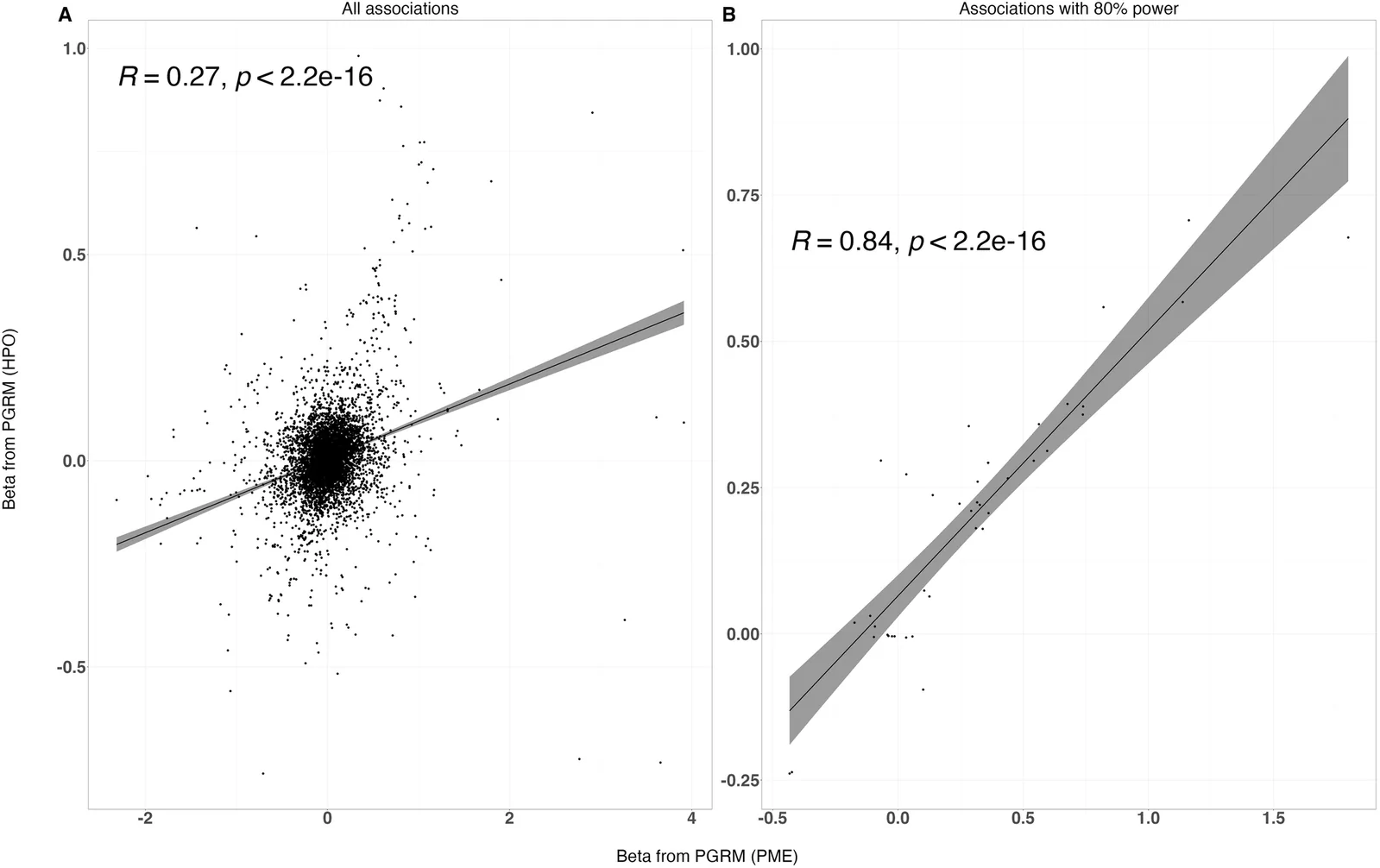
Correlation of established genotype-phenotype associations between PME and HPO. **A** Scatterplot of genotype-phenotype associations comparing β coefficients from phenome-wide association studies (PheWAS) in the patient-mediated EHR cohort (PME; *x*-axis) and the healthcare provider organization-provided EHR cohort (HPO; *y*-axis). Each point represents a single association. The solid line indicates the linear regression fit, with the shaded area representing the 95% CI. Spearman correlation coefficient (*R*) and *P* value are shown. **B** Scatterplot restricted to associations with at least 80% power in both cohorts, demonstrating stronger correlation between PME and HPO effect estimate.

We tested replication of 5727 previously established genotype-phenotype associations from the PGRM catalog^17^. Each association was evaluated in a trans-ancestry analysis combining all genetic ancestral groups as recommended by PGRM authors^17^, as well as in each genetic ancestral group in which the association was originally discovered, yielding a total of 10,999 and 6482 association tests in HPO and PME, respectively. In the trans-ancestry analysis, 5469 and 3565 associations were tested in HPO and PME, respectively. Across all groups, 5399 (49.1%) and 381 (5.9%) associations were replicated in the HPO and PME cohorts, respectively. In the trans-ancestry analysis, 2940 (53.8%) and 208 (5.8%) were replicated. Notably, 81 of the replicated associations in PME were not replicated in the HPO cohort. Among associations with adequate statistical power ( ≥ 80%), replication rates in the HPO cohort were 66.9% (2302/3442) in the trans-ancestry analysis, 75.9% (1517/1998) in EUR, 62.5% (20/32) in AFR, and 72.0% (18/25) in AMR. In the PME cohort, 28 associations were adequately powered in the trans-ancestry analysis, with a replication rate of 46.4% (13/28), and 13 in EUR, with a replication rate of 30.8% (4/13). No associations were sufficiently powered in other ancestry groups in PME. When examining the correlation of effect sizes for all 6474 shared associations pooled across the trans-ancestry and European analyses (Fig. 4), we observed a moderate correlation (*R* = 0.27). When restricting to associations adequately powered in both cohorts (*n* = 41), the correlation substantially improved (*R* = 0.84, *P* < 0.001), indicating strong concordance for well-powered genetic associations. All genotype-phenotype association results are presented in Supplementary Table S4.

### Sensitivity analysis

We performed stratified prevalence comparisons by self-reported race and ethnicity and state of residence within each EHR source (HPO vs PME). Within racial/ethnic strata, the number of phecodes tested (cases >20 in either cohort): 858 (White), 78 (Hispanic), and 66 (Black); the enrichment pattern was consistent across all strata (median PR: 5.4 [White], 5.4 [Hispanic], and 3.6 [Black]), and race/ethnicity-stratified PRs were highly correlated with the overall estimates (log-transformed PR Pearson *R* = 0.94, 0.82, and 0.84 for White, Hispanic, and Black strata, respectively). State-level comparisons were similarly limited to 72 phecodes (≥20 cases in both groups in ≥5 states); the median PR was 4.1, and state-level PRs were correlated with the overall estimates (log-transformed PR Pearson *R* = 0.81). Results of prevalence are included in Supplementary Tables S1 and S5.

After 1:1 propensity score matching, 19,703 HPO and 19,703 PME participants were retained, with all standardized mean differences at or below 0.05 (Supplementary Table S6). The prevalence enrichment in HPO relative to PME persisted after matching (median prevalence ratio 6.34, compared with 5.59 before matching across all phenotypes), and only 30.1% of phecodes moved closer to equal prevalence. The correlation between unmatched and matched log-transformed PRs was high (Pearson *R* = 0.920, Supplementary Table S7), indicating that the relative ranking and magnitude of disease enrichment in HPO were largely unchanged by matching on participant characteristics.

PheWAS of the same 10 diseases in the matched cohorts yielded 16,247 shared associations. Spearman correlations of comorbidity betas between matched HPO and PME across all shared associations ranged from 0.25 (colorectal cancer) to 0.41 (diabetes). When restricted to phenotypes with sufficient case counts (*n* > 100), correlations improved substantially: diabetes (*R* = 0.69), COPD (*R* = 0.63), IHD (*R* = 0.61), low back pain (*R* = 0.51), lupus (*R* = 0.41), cystic fibrosis (*R* = 0.42), lung cancer (*R* = 0.35), dementias (*R* = 0.35), multiple sclerosis (*R* = 0.29), and colorectal cancer (*R* = 0.26). These correlations were stable compared with the unmatched analysis, with 8 of 10 diseases showing changes within ±0.07 after matching. Full results of the matched PheWAS analyses, including per-disease odds ratios and confidence intervals for each cohort (original HPO, original PME, and matched HPO), are provided in Supplementary Table S3. The correlation plots are found in Supplementary Fig. S3.

In the matched cohorts, a total of 9895 and 6482 association tests were conducted across ancestry groups in matched HPO and PME, respectively. The overall PGRM replication rate across ancestry groups was 7.8% (767/9895) in matched HPO and 5.9% (381/6482) in PME (Fisher’s exact test OR = 1.35, *P* = 4.1 × 10⁻⁶), a substantial narrowing from the 8.3-fold difference observed before matching (49.1% versus 5.9% across ancestry groups). Among adequately powered associations in each cohort, the powered replication rate in the trans-ancestry analysis was 43.2% (54/125) in matched HPO and 46.4% (13/28) in PME; in the European ancestry analysis, rates were 67.2% (45/67) and 30.8% (4/13), respectively. When further restricting to associations adequately powered in both matched cohorts in the trans-ancestry analysis, replication rates were equivalent at 46.4% each (13/28), with strongly concordant effect sizes (*R* = 0.833). In the European ancestry subset, 8 of 13 associations powered in both cohorts were replicated in matched HPO (61.5%) versus 4 of 13 in PME (30.8%). Pooling the 41 powered associations across the trans-ancestry and European ancestry analyses, effect sizes remained strongly concordant (*R* = 0.76, *P* = 6.9 × 10⁻⁹). Additional details of results can be found in Supplementary Table S4.

## Discussion

This study provides a comprehensive comparison of PME data donation with traditional HPO-sourced data within *All of Us*. Our findings demonstrate that both sources of EHR data have utility for research, with PME data adding value to the pool of participants with EHRs and replicating known phenotype-phenotype and genotype-phenotype associations with effect sizes similar to HPO-provided data. Results from propensity score-matched analyses further confirmed that differences in genomic replication rates were largely attributable to sample size and cohort composition rather than intrinsic data quality. PME data captured longer observational periods and enabled patient-centered longitudinal health records for individuals who would otherwise not have EHRs. Nonetheless, fundamental differences in cohort composition and statistical power for discovery between these two data sources should be considered in the design and interpretation of studies utilizing *All of Us* and similar resources.

The lower disease prevalence observed in PME compared to HPO has several potential explanations. First, participants without HPO-sourced EHR data were most likely enrolled digitally. As such, they might represent a healthier subset of the population with greater digital health literacy who primarily have chosen to enroll in *All of Us* in response to digital engagement. This is consistent with our finding that the PME cohort had higher rates of insurance coverage and educational attainment. Second, patient portals may not capture all healthcare encounters by an individual and may be biased toward outpatient care instead of hospital care^18^. Third, HPO-based recruitment may preferentially enroll individuals with more extensive healthcare needs who have more frequent encounters with the healthcare system. The consistent pattern of higher disease prevalence across multiple categories of diseases, particularly for chronic diseases in both *All of Us* cohorts compared to national estimates, aligns with previous findings^13,14,19^. This overrepresentation of disease in HPO vs PME may result from the healthcare-based recruitment strategies employed by *All of Us*. The persistence of this enrichment after propensity score matching on demographic, socioeconomic and EHR documentation characteristics, and its consistency across racial/ethnic groups and states of residence, further supports that the prevalence gap reflects a healthier population in PME rather than differences in participant demographics, although the possibility of potentially shallower clinical documentation through patient-mediated records might not be fully eliminated. The reason for a higher disease prevalence of encephalitis among those with PME is less clear. It could represent a stronger response rate to join *All of Us* among those with a history of this illness upon hearing about *All of Us* or a greater likelihood of coming in contact with *All of Us* messages from other patients, advocacy groups, or social media.

Despite differences in prevalence, we replicated known disease-phenotype associations of commonly studied diseases in both cohorts. Among phenotypes with larger case numbers, the effect size estimates show strong correlations between the two cohorts. These correlations were stable after 1:1 propensity score matching and were comparable to or exceeded those previously observed between *All of Us* and UK Biobank^14^, a well-established and widely used population-based cohort. These findings are reassuring for researchers using either data source.

The substantially lower replication rate for established genotype-phenotype associations in PME data compared to HPO likely reflects reduced sample size and loss of statistical power rather than absence of true signal. This interpretation is directly supported by our propensity score–matched analyses: after equalizing sample sizes and key demographic characteristics through 1:1 matching, the replication gap narrowed dramatically. Among adequately powered associations, matched HPO and PME achieved comparable replication rates, and the powered replication rate in matched HPO was in fact numerically lower than in PME in the trans-ancestry analysis. Effect size estimates for well-powered associations were strongly correlated between the two data sources, and this concordance was maintained after matching. The residual overall difference in replication rates likely reflects the greater number of testable associations in matched HPO (9895 versus 6482), attributable to more phenotypes documented, rather than a per-association accuracy advantage. Additionally, approximately one in five associations replicated in PME were not replicated in the larger HPO cohort, suggesting that each data source may capture distinct aspects of phenotypic variation that contribute uniquely to genetic association detection.

These findings have important implications for researchers using *All of Us* and similar datasets. When integrating data from both sources, it is essential to account for systematic differences in disease prevalence and participant characteristics, which may require stratification by EHR source or inclusion of source as a covariate in analytic models^20^. In addition, more stringent definitions that incorporate supporting evidence beyond diagnosis codes can help harmonize case definitions across sources^21^. Power limitations should also be considered in genetic analyses: although both sources can replicate established associations when sufficiently powered, the smaller sample size and lower disease prevalence in the current PME cohort should be considered in study design and statistical testing^22^.

This study has several additional limitations. First, we focused on disease presence as defined by diagnosis codes and did not evaluate other clinical parameters such as laboratory values, medications, or procedures, which can miss some of the conditions. Second, the small sample size of the PME cohort limited statistical power for comparison and genetic analyses. We expect that the future release of EHR data, including data from both sources in the same set of participants, will help elucidate the observed differences. Importantly, these results support the validity of combining data from multiple sources to help address EHR data fragmentation issues.

In summary, our study demonstrates that both PME and HPO-sourced EHR data capture meaningful phenotypic signals suitable for large-scale association studies, with PME offering a valuable complement that extends the reach of *All of Us* to participants beyond traditional healthcare provider networks. The observed differences in disease prevalence between sources underscore the importance of understanding and accounting for data source characteristics when designing studies that combine these cohorts. We anticipate that these findings will help guide researchers in leveraging the full breadth of EHR sources available in *All of Us* and may inform similar efforts in other large-scale research programs integrating multiple streams of health data.

## Methods

### Study population

All analyses were conducted in accordance with U.S. federal regulations for the protection of human subjects (45 CFR 46) and the *All of Us* Data User Code of Conduct and the Data and Statistics Dissemination Policy. Access to data was restricted to authorized investigators who completed the *All of Us* Responsible Conduct of Research training. The *All of Us* Institutional Review Board determined that research conducted using Controlled Tier data under the data-passport model does not constitute human-subjects research (Protocol 2021-02-TN-001); accordingly, institutional review board approval and additional human ethics or consent-to-participate declarations were not required.

We analyzed data from the Controlled Tier Dataset version 8 of *All of Us*, which included participants enrolled between May 2017 and October 2023. Detailed descriptions of the *All of Us* cohort, recruitment strategies, and data collection methods have been published elsewhere^5,9^. Briefly, *All of Us* enrolls participants aged 18 years and older from different backgrounds across the United States^9^. Participants provide informed consent, complete surveys, undergo physical measurements, provide biospecimens, and may share their EHRs. Details of the consent contents and process have been described previously^23^.

We included participants who had at least one diagnosis code (ICD-9-CM or ICD-10-CM) in their EHR data. For this analysis, we defined two cohorts based on the source of EHR data: The PME cohort (*n* = 19,703) included participants who provided access to their EHRs through patient portal linkages or other patient-mediated mechanisms; The HPO cohort included 373,887 participants^18^. Details of the HPO sites can be found at https://www.joinallofus.org/health-care-provider-organizations.

### Data collection and harmonization

HPO data were obtained by structured extraction from EHR systems at HPOs participating as *All of Us* enrollment sites, including encounter-based diagnoses, procedures, medications and laboratory measurements from inpatient and outpatient care. PME data were obtained through participant-linked patient portals using FHIR-based data exchange and could include records from any health system where the participant received care, including systems outside the *All of Us* network. The two data streams capture distinct ascertainment pathways. HPO data provide institution-level longitudinal completeness within participating systems, whereas PME data can extend capture across otherwise unlinked care settings. In this release, participants were assigned to a single source, such that HPO and PME cohorts were mutually exclusive.

Within both cohorts, EHR data on transportation-related injuries, some pregnancy and neonatal outcomes were excluded to minimize the risk of re-identification. Participants completed surveys on demographic characteristics and many other factors, including sex, race, ethnicity, highest education attained, annual household income, and health insurance status, which were collected through surveys at enrollment^24^. Information on EHR length and first documented age in EHRs was derived from the EHR data. We included first age in EHRs-defined as the participant’s age at the date of their earliest recorded diagnosis code as a covariate rather than age at enrollment, as it better captures the start of the observable clinical history and accounts for the retrospective nature of linked EHR data, which often predates program enrollment. EHR length was calculated as the difference in years between the earliest and latest recorded diagnosis codes for each participant.

### Genomic data

Genomic data are provided primarily as short-read whole-genome sequencing (srWGS); microarray genotyping was generated mainly for quality control and was not imputed, with srWGS and array data overlapping in >92% of genotyped participants. srWGS was available for 83.2% of HPO and 35.2% of PME participants (array genotyping: 88.9% and 40.9%). Because *All of Us* continues to generate srWGS for all participants, we restricted genotype-based analyses to participants with srWGS rather than add an interim imputation step. Accordingly, the PGRM replication analyses used only participants with srWGS, whereas the prevalence and phenotype–phenotype association analyses required no genomic data and used the full EHR cohorts.

### Phenotyping

To compare disease prevalence across the phenome, we mapped ICD-9-CM and ICD-10-CM diagnosis codes to standardized phenotypes using phecodes (version X)^25^. Phecodes group related ICD codes into clinically meaningful categories, allowing for comprehensive phenome-wide analyses. For each phenotype, participants with at least two occurrences of the corresponding phecode in their EHR were classified as cases, consistent with established phenotyping methods^26^. The validity and reproducibility of phecode-based phenotyping have been demonstrated in previous studies^10,26,27^. A total of 2840 shared phenotypes defined by phecodes were identified in both cohorts.

For comparison with national disease prevalence, we mapped ICD codes to diseases defined in the Global Burden of Diseases (GBD) study^15^. We used the average estimates for the US population from 2018 to 2021 to align with the timeframe of the *All of Us* enrollment and data curation. A total of 292 and 219 GBD phenotypes with at least 20 cases were identified in HPO and PME cohorts, respectively.

### Statistical analysis

We compared baseline characteristics between the PME and HPO cohorts using chi-square tests for categorical variables and *t*-tests for continuous variables. Values are *n* (%) for categorical variables and mean (SD) for continuous variables. *P* values were calculated using chi-square tests for categorical variables and *t*-tests for continuous variables.

For each phecode-defined phenotype, we calculated prevalence (with 95% confidence intervals) in both cohorts. Prevalence ratios (PRs) were computed by dividing the prevalence in the HPO cohort by that in the PME cohort.

We performed phenome-wide association studies (PheWAS) for 10 diseases that have been well-studied, including 7 common diseases (IHD, lung cancer, COPD, Alzheimer’s disease and other dementias, colorectal cancer, type 2 diabetes, and low back pain), which are leading causes of mortality or morbidities in the US^13^, and 3 less common diseases including one rare disease (multiple sclerosis, lupus, and cystic fibrosis)^14^. For common diseases, we performed the PheWAS using logistic regressions adjusted for age, sex at birth, self-reported race or ethnicity, length of EHR records, income, and education. For multiple sclerosis, lupus, and cystic fibrosis, we used Firth logistic regressions adjusted for the same covariates. We calculated Spearman correlation coefficients of effect sizes (beta coefficients) estimated from regressions between the PME and HPO cohorts for each disease. We considered an association replicated if it had a *P* < 0.05 and a direction of effect consistent with previous literature. We applied PheWAS in its general formulation, with a phenotype (each index disease) rather than a genetic variant as the exposure, an approach well established for non-genetic exposures^26,28^.

To assess replication of genotype-phenotype associations, we used the phenotype-genotype reference map (PGRM, v 0.1.0), a curated set of 5879 genetic associations from 523 genome-wide association studies (GWAS), specifically designed to enable systematic replication studies across different biobanks^17^. PGRM associations are categorized by the genetic ancestry of their original discovery cohorts, comprising African ancestry (AFR; number of associations (*n*) = 49), East Asian ancestry (EAS; *n* = 1,215), European ancestry (EUR; *n* = 4,568), Admixed American (AMR; *n* = 30), and South Asian ancestry (SAS; *n* = 17). We evaluated the replicability of associations using data across each genetic ancestry, including all ancestries combined (trans-ancestry) as suggested^17^. We tested these associations in both cohorts, using logistic regression models adjusted for age, sex, and principal components derived from genetic data. We considered an association replicated if it had a *P* < 0.05 and a direction of effect consistent with the original GWAS. We computed the Spearman correlation of effect sizes between cohorts for all tested associations and for the subset with adequate statistical power ( ≥ 80%).

All statistical significance testing was 2-sided. All statistical analyses were conducted within the *All of Us* workbench with Python (version 3.9.10) or R (version 4.2.0). For phenome-wide comparisons, we used *P* < 1.8 × 10^−5^ as the statistically significant threshold, based on a Bonferroni correction of 2840 phenotypes. The scripts will be available to approved researchers through the *All of Us* research workbench upon request.

### Sensitivity analysis

To assess whether the observed prevalence differences between HPO and PME were driven by demographic composition, we computed prevalence ratios stratified by self-reported race/ethnicity and state of residence. For each phecode, prevalence was calculated separately within each EHR source and stratum, using participants in that stratum as the denominator. Prevalence ratios (HPO:PME) were then compared across strata.

To compare the HPO and PME cohorts independent of differences in participant characteristics, we performed 1:1 nearest-neighbor propensity score matching. Propensity scores were estimated using logistic regression with EHR source (HPO versus PME) as the dependent variable and the following covariates: first age in EHR, length of EHR record, sex at birth, self-reported race and ethnicity, educational attainment, annual household income, health insurance status, availability of whole genome sequencing data, and smoking status. Matching was performed without a caliper to ensure retention of all 19,703 PME participants, each paired with one HPO participant. Covariate balance was assessed using standardized mean differences, with a threshold of less than 0.1 considered well balanced. Using the matched cohorts, we repeated the PGRM replication analysis and the disease-specific PheWAS for all 10 index diseases.

To evaluate the impact of matching on disease prevalence, we computed phecode-level prevalence ratios (HPO/PME) before and after matching, overall and stratified by self-reported race and ethnicity and state of residence. Prevalence ratios were compared using paired Wilcoxon signed-rank tests, and correlation of log-transformed prevalence ratios before and after matching was assessed using Pearson correlation coefficients.

## Supplementary information

Supplementary information

Table S1 Please use the uploaded Table S1 for this information.

Table S2

Table S3

Table S4

Table S5

Table S6

Table S7

## Data Availability

Deidentified participant data used in this study are available through the *All of Us* Research Program Researcher Workbench (https://researchallofus.org) as a shared, cloud-based research workspace. Access is available to any approved *All of Us* researcher worldwide and is granted in accordance with *All of Us* data access policies. All data at the summary level supporting results reported in this study are included in the Supplementary Tables S1–7 and Supplementary Figures S1–2.

## Code availability

Access is available to approved *All of Us* researchers through the *All of Us* research workbench.
