# Supplementary material for: Robust replication of associations across patient-mediated and provider-sourced EHR data in the All of Us research program
Source: NPJ Digit Public Health. 2026 Sep 7;1(1):31. doi: 10.1038/s44482-026-00032-8 (PMC13549936; doi:10.1038/s44482-026-00032-8)
Supplement: Supplementary file 1 [file 44482_2026_32_MOESM1_ESM.pdf]

Supplementary information

Supplemental Figures and Tables

Supplemental Figures

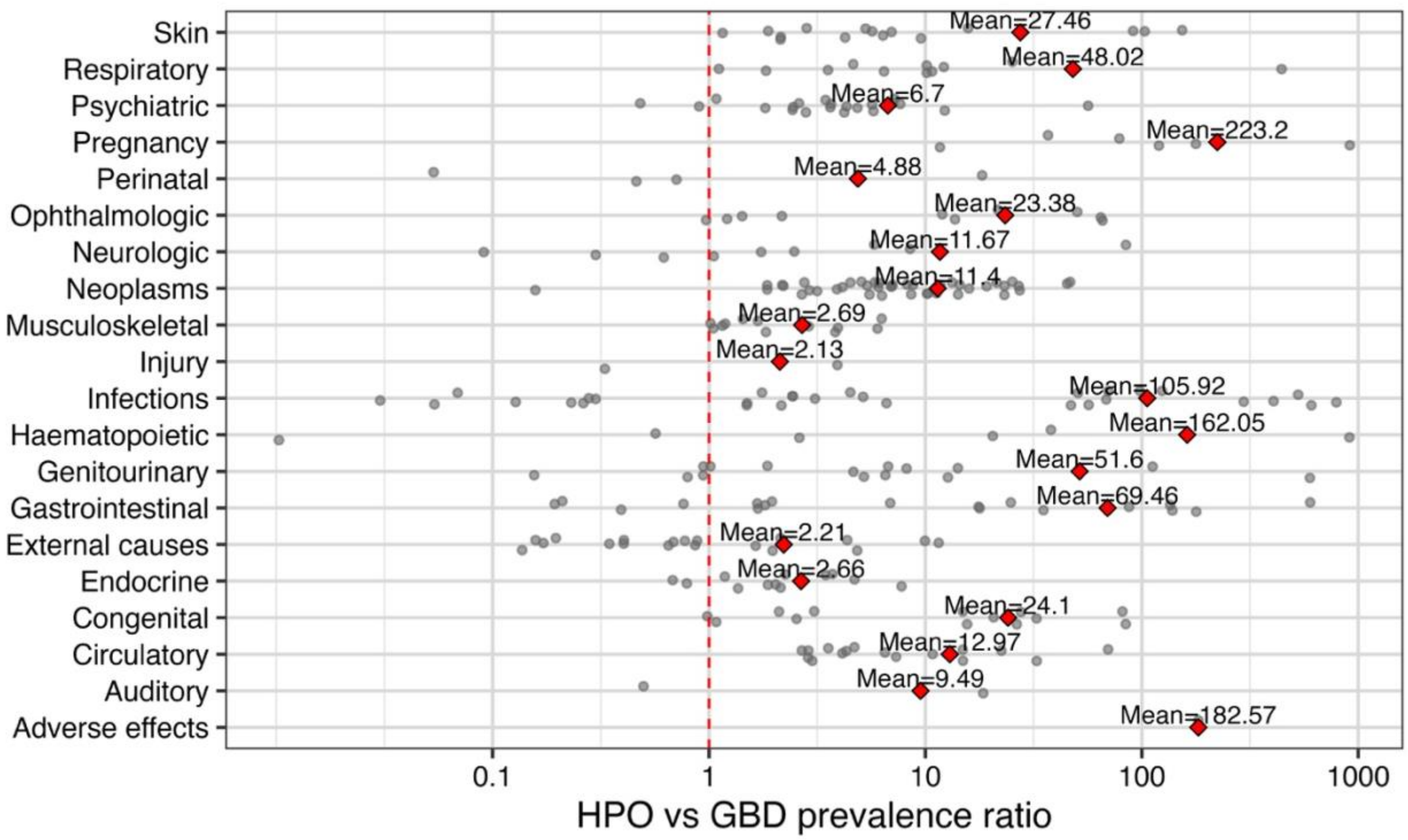

Supplemental Figure S1. Disease prevalence ratios comparing the healthcare provider organization EHR (HPO) cohort within *All of Us* with the Global Burden of Disease (GBD), US, 2018-2021. Scatter plot showing prevalence ratios for individual conditions grouped by disease category (y-axis), comparing participants in the HPO cohort with those in GBD. Each grey point represents a condition-level prevalence ratio plotted on a log scale (x-axis). Red diamonds indicate the mean prevalence ratio within each disease category, with annotated mean values. The vertical dashed line at ratio = 1 denotes equal prevalence between HPO and GBD. Estimates of prevalence of diseases of the US general population and ICD-cause mapping file were downloaded from the Institute for Health Metrics Websites (https://vizhub.healthdata.org/gbd-results/). Source: Institute for Health Metrics and Evaluation. Used with permission. All rights reserved.

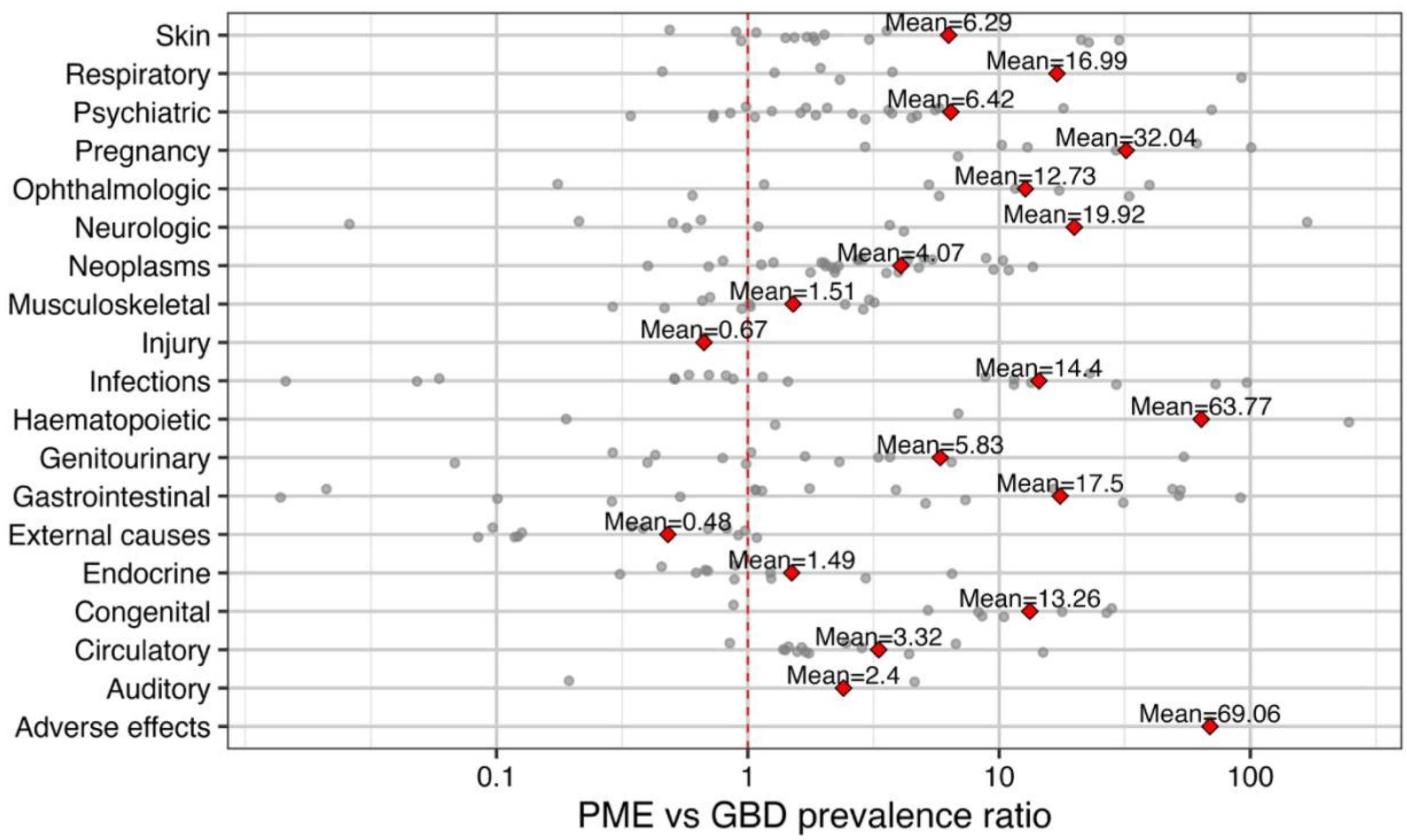

Supplemental Figure S2. Disease prevalence ratios comparing the participant-mediated EHR (PME) cohort within *All of Us* with the Global Burden of Disease (GBD), US, 2018-2021. Scatter plot showing prevalence ratios for individual conditions grouped by disease category (y-axis), comparing participants in the PME cohort with those in GBD. Grey points represent condition-level prevalence ratios on a log scale (x-axis). Red diamonds denote category-specific mean prevalence ratios, with annotated mean values. The vertical dashed line at ratio = 1 indicates no difference in prevalence between PME and GBD. Estimates of prevalence of diseases of the US general population and ICD-cause mapping file were downloaded from the Institute for Health Metrics Websites (https://vizhub.healthdata.org/gbd-results/). Source: Institute for Health Metrics and Evaluation. Used with permission. All rights reserved.

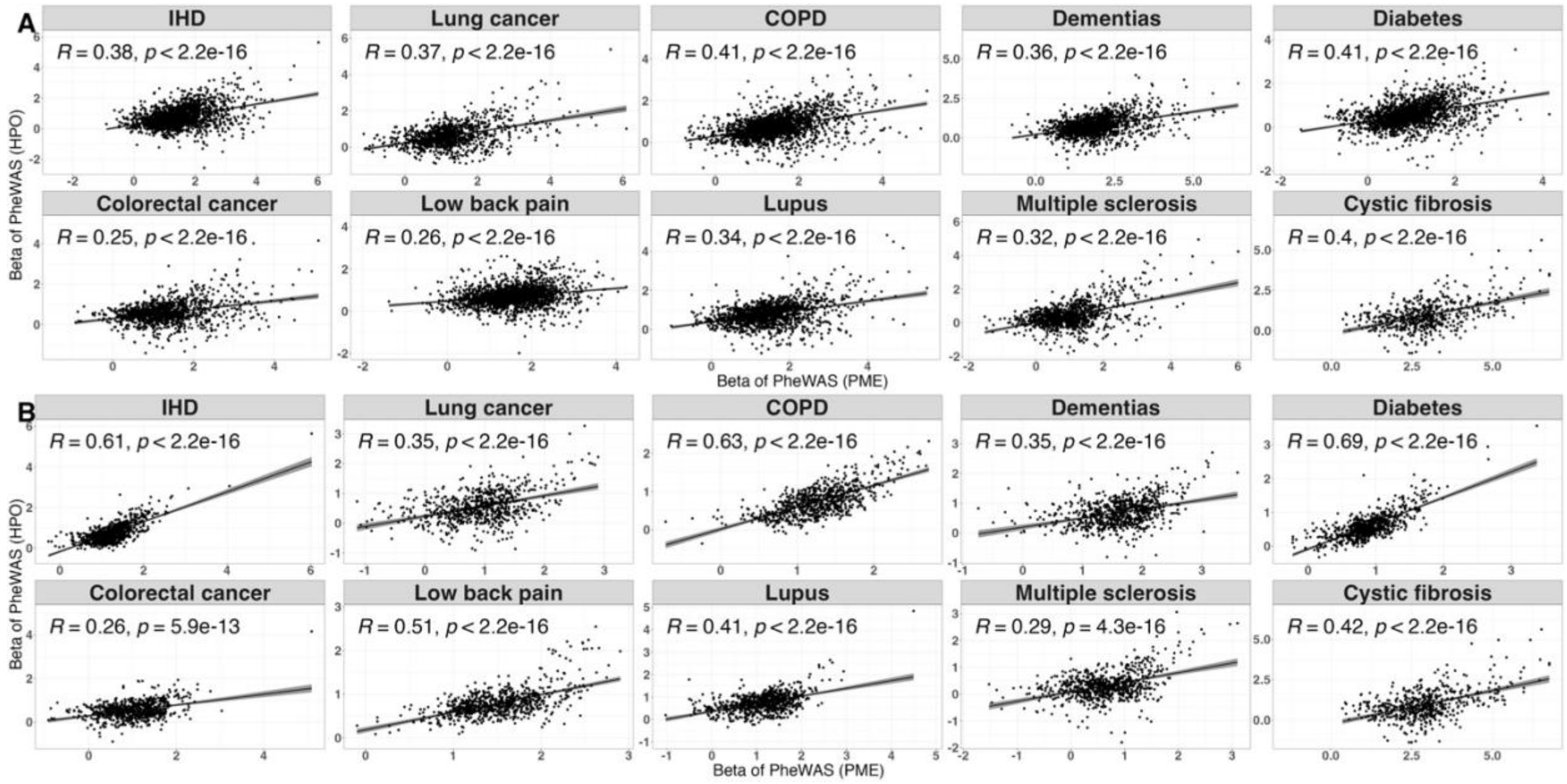

Supplemental Figure S3 Correlation of Phenotype-Phenotype Associations Between PME and the 1:1 propensity-score matched HPO cohort.

A. Scatterplots show the association between phecode effect size estimates (β coefficients) obtained from phenome-wide association studies (PheWAS) in the participant-mediated EHR cohort (PME; x-axis) and the 1:1 propensity-score matched healthcare provider organizations-provided EHR cohort (HPO; y-axis). Each dot represents a phenotype-phenotype association; solid lines indicate linear regression fits with 95% CIs (shaded). Panels display selected diseases, with Spearman correlation coefficients (R) and corresponding P values reported. B. Scatterplots show β coefficients obtained from associations with at least 100 cases in both cohorts. the participant-mediated PME; x-axis and HPO; y-axis. Each dot represents a phenotype-phenotype association; solid lines indicate linear regression fits with 95% CIs (shaded). Panels display selected diseases, with Spearman correlation coefficients (R) and corresponding *P* values reported.

Supplemental Tables

Supplemental Table S1. Phenome-wide comparison of disease prevalence in PME and HPO cohorts.

Supplemental Table S2. Comparison with the Global Burden of Disease (US, 2018-2021).

Supplemental Table S3. Associations of 10 diseases with phenotypes across the phenome in PME and HPO cohorts and in the 1:1 propensity-score matched HPO cohort.

Supplemental Table S4 Associations of established genotype-phenotype associations in HPO and PME cohorts and in the 1:1 propensity-score matched HPO cohort.

Supplemental Table S5. Phenome-wide comparison of disease prevalence in PME and HPO cohorts by race and ethnicity and state of residence.

Supplemental Table S6. Baseline characteristics after 1:1 propensity-score matching.

Supplemental Table S7. Phenome-wide comparison of disease prevalence in PME and the 1:1 propensity-score matched HPO cohort.
